# Chronic Polyphenon-60 or Catechin Treatments Increase Brain Monoamines Syntheses and Hippocampal SIRT1 LEVELS Improving Cognition in Aged Rats

**DOI:** 10.3390/nu12020326

**Published:** 2020-01-26

**Authors:** Margarita R. Ramis, Fiorella Sarubbo, Silvia Tejada, Manuel Jiménez, Susana Esteban, Antoni Miralles, David Moranta

**Affiliations:** 1 Laboratory of Neurophysiology, Biology Department, University of Balearic Islands (UIB), Ctra. Valldemossa Km 7,5 E-07122 Palma de Mallorca, Spain; margaramis87@gmail.com (M.R.R.); mariafiorella.sarubbo@hsll.es (F.S.); silvia.tejada@uib.es (S.T.); m.jimenez1@estudiant.uib.cat (M.J.); susana.esteban@uib.es (S.E.); amiralles@uib.es (A.M.); 2 Research Unit, Hospital Universitario Son Llàtzer, Health Research Institute of Balearic Islands (IdISBa), Carretera de Manacor Km 4, E-07198 Palma de Mallorca, Spain; 3 Oncología quirúrgica procedimientos mínima invasión, Health Research Institute of Balearic Islands (IdISBa), Ctra. Valldemossa 79, E-07120 Palma de Mallorca, Spain; 4 CIBERON (Physiopathology of Obesity and Nutrition), 28029 Madrid, Spain

**Keywords:** brain aging, memory, green tea, catechin, brain monoamine synthesis, SIRT1, RBAP46/48, NF-κB

## Abstract

Polyphenolic compounds from green tea have great interest due to its large CONSUMPTION and therapeutic potential on the age-associated brain decline. The current work compares a similar dose regimen of a whole-green-tea extract and catechin in old rats over the course of 36 days. Results showed a significant improvement in visuo-spatial working memory and episodic memory of old rats after polyphenolic compounds administration assessed by behavioral tests. No effects were observed on the age-associated motor coordination decline. Statistically, results were correlated with significant improvements, mainly in hippocampal and striatal noradrenergic and serotonergic systems, but also with the striatal dopaminergic system. Both polyphenolic treatments also reverted the age-associated reduction of the neuroinflammation by modulating protein sirtuin 1 (SIRT1) expression in hippocampus, but no effects were observed in the usual reduction of the histone-binding protein RBAP46/48 protein linked to aging. These results are in line with previous ones obtained with other polyphenolic compounds, suggesting a general protective effect of all these compounds on the age-associated brain decline, pointing to a reduction of the oxidative stress and neuroinflammatory status reduction as the leading mechanisms. Results also reinforce the relevance of SIRT1-mediated mechanism on the neuroprotective effect and rule out the participation of RBAP46/48 protein.

## 1. Introduction

The increasing longevity of the world population has produced an on-going epidemiological transition, where noncommunicable diseases emerge as major threats to the public health systems [1]. These diseases are also related to disability, dependency, and long-term care needs, especially in the case of dementia. In low- and middle-income countries, the prevalence of dementia is higher than in high-income ones, suggesting that environmental factors, and particularly lifestyle, are relevant to deal with this big challenge for the public health [1].

Aging induces important brain physiological changes; among them, synaptic dysfunction may be the main cause of the cognitive alterations during normal aging. Oxidative stress environment is believed to be one of the main factors of this age-associated brain disfunction, induced by reduced endogenous antioxidant levels and increased reactive oxygen species (ROS) production, linked to a chronic low-grade neuroinflammatory status [2,3,4]. Dietary antioxidants, with special relevance of polyphenols due to their availability in the diet, have been shown to limit some effects of aging, particularly brain aging, due to their capacity to cross the blood–brain barrier [5,6,7]. Among polyphenol-rich products, the green tea extracts (made from *Camellia sinensis* leaves) have drawn attention due to its large consumption worldwide as an infusion. Green tea extracts are rich in flavonoid compounds, mainly catechins (around 30–42% of solid extract weight) [8,9,10]. In addition, polyphenon-60 is a catechin extract from green tea, composed of a mixture of the main active polyphenols components of green tea [11]. Green tea extracts intake has been related to a variety of beneficial health effects, including neuroprotective ones (see [10,12,13] for review), and with special therapeutic potential during brain aging [14,15,16]. In this sense, several epidemiological studies have associated tea consumption (sometimes regardless of whether it is green or black tea) not only with a better cognitive performance [17,18,19], but also with other sources of catechins [20].

Many brain functions are modulated by monoaminergic systems. Thus, cognitive and motor impairments associated with aging have been related to a marked age-associated decline of these systems, which is mainly observed in cognitive-related brain regions [21,22]. Additionally, protective effects of antioxidant compounds (including some polyphenolic compounds) have been related to the recovery or protection of the monoaminergic systems [5,6,23,24]. Despite this fact, the protective effect of the green tea exposure on these monoaminergic systems has not been reported yet. In addition, molecular mechanisms underlying neuroprotective properties of polyphenolic compounds have not yet been well elucidated (not only for the ones present in green tea extracts but also for other polyphenols) [25]. In this sense, several molecular mechanisms have been explored to explicate catechin neuroprotective properties. Among others, epigenetic mechanisms seem to play a relevant role [26,27]. In this regard, neuroprotective properties of other polyphenols have been associated to the NAD-dependent histone deacetylase, sirtuin 1 (SIRT1) protein; since this protein shows a marked age-related reduction in brain, mainly in the hippocampus [6,25,28]. However, the effect of green tea extract on this protein has not been investigated deeply, at least in vivo with a focus on the brain [29,30]. SIRT1 has a relevant role regulating brain functions such as plasticity and memory [25,31]. Among SIRT1-modulated signaling pathways, many of the SIRT1-associated neuroprotective effects can be attributed to NF-κB signaling modulation, affecting proinflammatory responses and cell survival [2,25,32,33]. Finally, another protein that modulates histone acetylation patterns, the histone-binding protein RBAP46/48, has also been associated with age-related memory loss [34,35]; its role in the neuroprotective effects of polyphenolic compounds has not been investigated.

Thus, the present work aims to study the effect of green tea extract and catechin on brain monoaminergic systems, SIRT1 and RBAP46/48 hippocampal levels, and in the cognitive status of old rats.

## 2. Materials and Methods

### 2.1. Animals, Drugs, Reagents, and Treatments

Old male Sprague-Dawley rats (18 months; 640 ± 5 g weight; *n* = 16; Charles River, Spain) were housed individually in standard cages under controlled environmental conditions (20 ± 2 °C; 70% humidity, and 12-h light/dark cycle, lights on at 08:00) with free access to standard food (Panlab A04, Spain) and tap water. All procedures were performed during the light period and in accordance with the European Convention for the Protection of Vertebrate Animals used for Experimental and other Scientific Purposes (Directive 86/609/EEC) and approved by the Bioethical Committee of the University (approval file number 2014/05/AEXP). Old male rats were chronically treated, over the course of 28 days with 20 mg/kg·day (i.p.) of polyphenon-60 (*n* = 6; green tea extract from Sigma-Aldrich containing a minimum of 60% of total catechins) or (+)-catechin (*n* = 5; (2R,3S)-2-(3,4-dihydroxyphenyl)-3,4-dihydro-1(2H)-benzopyran-3,5,7-triol from Sigma-Aldrich) dissolved in corn oil (Sigma-Aldrich). The vehicle (corn oil, 1 mL/kg·day, i.p., *n* = 6) was used in the old control group. Initially, the catechin group was also constituted by *6* animals but one animal from the catechin group died after 1 week of treatment. The cause of death could not be determined. A group of naive young rats (3 months of age, around 350 ± 6 g weight; *n* = 6; Charles River) were used as reference to compare with old animals. Cognitive abilities were monitored during the chronic treatments (see following sections) and although an improved tendency was observed, some behavioral test results do not reach statistical significance (see results section). For this reason, it was decided to continue the treatment one additional week while increasing the dose to 40 mg/kg·day. After this additional week, a significant improvement in cognitive abilities due to treatments was observed (see results sections). Rats were sacrificed by decapitation the following day after finishing the behavior analysis, at 08:00 (during dark/light change). In order to analyze monoamines synthesis, all rats received a single administration of NSD 1015 (which is a central aromatic amino acid decarboxylase inhibitor) (100 mg/kg, i.p.) 30 min before sacrifice (under red light) to measure the in vivo activity of tryptophan hydroxylase (TPH) and tyrosine hydroxylase (TH), through accumulation of 5-hydroxytryptophan (5-HTP) and dihydroxyphenylalanine (DOPA), respectively (see below). Brains were quickly removed and dissected on an ice-cold plate so as to separate the hippocampus and the striatum (which includes the caudate nucleus and the putamen) from both cerebral hemispheres, and then they were frozen in liquid nitrogen immediately and stored at −80 °C until analysis.

Drugs and reagents were purchased from Sigma-Aldrich (a part of the Merck group, Germany), unless otherwise indicated.

### 2.2. Behavioral Tests

Behavioral tests were conducted between 09:00 and 13:00 h. The animals were analyzed individually, alternating animals of the different experimental groups to avoid possible chronobiological masked effects. Animals were placed in the experimental room 30 min before tests for familiarization. Tests were performed every 15 days during chronic treatments to monitor cognitive and motor ability evolution. In each session, the radial maze test was the first test performed, the following day the novel object recognition test was carried out, and the rotarod test was performed the third day. Some days before performing the tests, animals were subjected to familiarization. Familiarization sessions were carried out once the tests were completed when they matched with tests. Additionally, a fasting period was also necessary for the radial maze test (see following section for specific information of each test).

#### 2.2.1. Radial Maze Test

Radial maze test was used to test spatial working memory in rats, as previously reported [6,36]. The eight-arm radial maze (Panlab) consisted of an octagonal central platform (30 cm diameter) with eight equally spaced radial arms (70 cm long and 10 cm wide) with visual clues at arm entrance consisting of different shapes of the same color. The maze was set in an experimental room with several external visual cues. To test working memory, rats were allowed to make an arm choice to obtain small pieces of food pellets (located at the end of each arm, Panlab A04, Spain) until all eight arms were visited; in case rats did not finish the task, the maximum time given was 20 min. Animals were submitted to 48 h fasting before each radial maze test in order to achieve the same motivational level, as described previously [37]. The movements of the animals were monitored by means of a digital video tracking system (LE 8300 with software SEDACOM v 1.3, Panlab) and analyzed with the software SMART v 2.5 (Panlab). The sum of nonvisited arms and re-entry into arms was scored as working memory errors. Distance traveled was also recorded. The maze was cleaned with ethanol 70% between trials to eliminate any olfactory trail.

#### 2.2.2. Novel Object Recognition Test

The novel object recognition test is a method to measure episodic-like memory in rodents, which assesses their natural propensity to explore the novel object as their natural tendency to novelty [38]. It was performed in a white open field device (78 cm diameter cylinder with 60 cm high opaque wall). The test involved three phases: habituation, familiarization, and test phase [6,39]. In the habituation phase, each animal could freely explore the open field in the absence of objects for 10 min, daily, during 4 consecutive days. On day 5, each animal was placed in the apparatus and allowed to explore for 1 min for re-habituation. During the familiarization phase, animals were placed in the center of the apparatus with two identical objects (i.e., blue plastic cubes, 6 cm × 6 cm × 6 cm ) always placed in the same location by using velcro on the base of the objects (35 cm between them, 16 cm away from the walls). Animals were allowed to explore both objects until 10 min had elapsed. Object exploration was considered when the nose or mouth of the animal were in contact with the object. After a delay of 10 min, the test phase was conducted. So, the animals were placed back in the open field with one familiar object (the same as in the previous phase) and one novel object (i.e., red plastic 8 cm × 4 cm with bolus shape). The time spent exploring the objects was recorded until 10 min had elapsed. The objects had no natural significance for animals, since they are made of the same material in different shapes and had never been associated with reinforcement or location. Moreover, during the training session both objects were novel, so that the time spent on both objects should be similar, which was verified in each session. In this way, the test phase reflects the preference for novelty. Discrimination index (D.I.) was calculated following the equation:

D.I. = (time exploring novel object − time exploring familiar object)/total time exploring any object.

The objects and the device were cleaned with ethanol solution between trials. In addition, anxiety and exploratory drive of the animals were measured by recording the number of times that the rats passed through the different areas of the open field and the number of urinations and defecations.

#### 2.2.3. Motor Coordination in Rotarod Test

Motor ability and balance were evaluated on the rotarod treadmill device (Panlab). Animals were subjected to training sessions during 4 days prior to the test (one session/day) on a rotarod at a constant speed of 4 rpm until they achieved a stable performance. In the test phase, the rats were placed on the rotarod in acceleration mode (from 4 to 40 rpm over a period of 60 s) for recording the latency to fall. Each rat repeated the test five times, leaving some minutes for recovery between tests. The mean measured was used as reference of motor coordination.

### 2.3. TPH Activity (Synthesis of 5-HT) and TH Activity (Synthesis of DA and NA)

Transformation of tryptophan into 5-HTP is the limiting step in 5-HT synthesis which requires the TPH-2 isoform enzyme for the most part of the brain and TPH-1 isoform enzyme in the pineal gland [40]. Regarding catecholamine (NA and DA) synthesis, the limiting step is the transformation of tyrosine into DOPA which requires the TH enzyme. The in vivo activities of these rate-limiting enzymes were determined by measuring the accumulation of 5-HTP and DOPA within 30 min after inhibition of the aromatic L-amino acid decarboxylase by a maximally effective dose of NSD 1015 (3-hydroxybenzylhydrazine HCl, 100 mg/kg, i.p.). Accumulation of 5-HTP indicates 5-HT synthesis in all brain regions, whereas DOPA accumulation indicates synthesis of NA in the hippocampus and synthesis of DA in the striatum. This method also enables to quantify the pool of 5-HT, DA, or NA unaffected by recent synthesis and primarily stored within neurons. Furthermore, it also allows to determinate the levels of some monoaminergic metabolites that can reveal recent use of these neurotransmitters: 5-hydroxyindoleacetic (5-HIAA) and 3,4-dihydroxyphenylacetic acid (DOPAC) [5,6]. All these compounds (monoamines, precursors, and metabolites) were determined simultaneously by high-performance liquid chromatography (HPLC) with electrochemical detection. Frozen brain regions (one of the two hippocampus and striatum regions) were quickly weighted and homogenized with an Ultra-Turrax homogenizer (Type Tp 18/10, Janke and Kunkel, Germany) in 1 mL of cold 0.4 M HClO_4_, 0.01% K_2_EDTA and 0.1% Na_2_S_2_O_5_. Obtained homogenates were centrifuged at 40,000× *g* for 15 min at 4 °C. The resulting supernatant was filtered (0.45 µm pore diameter, Millex LH, Millipore) and aliquots were injected into the HPLC system on a reversed-phase column (Spherisorb S3 ODS1 C18; 3 µm particle size range) coupled to a Tracer ODS2 C18 precolumn (2–5 µm particle size range, Teknokroma, Spain) maintained at 35 °C. The mobile phase consisted of 0.1 M KH_2_PO_4_, 2.1 mM octane sulfonic acid, 0.1 mM K_2_EDTA, 2 mM NaCl, and 12% methanol (pH 2.7–2.8, adjusted with 85% H_3_PO_4_), which was pumped at a flow rate of 0.8 mL/min with a Waters M-600 controller solvent delivery system (Waters, Spain). The compounds were electrochemically detected by a cell with a glassy working carbon electrode with an applied oxidation potential of +0.75 V against an in-situ Ag/AgCl reference electrode (Waters M-2465 Electrochemical Detector). The output electric current was monitored by an interphase (Waters busSAT/IN Module) connected to a computer. The concentrations of the compounds in a given sample were calculated by interpolating the corresponding peak height into a parallel standard curve using the software Empower Pro (Waters).

### 2.4. Western Blot Analysis

The remaining hippocampus from animals was homogenized in 1:15 weight/volume of cold homogenization buffer (50 mM Tris-HCl, pH 7,5; 1 mM EDTA; 2% SDS) in the presence of a protease inhibitor cocktail (Pierce) with an Ultra-Turrax homogenizer (Type Tp 18/10, Janke and Kunkel, Germany) two times for 10 s each sample. Extracts were also sonicated two times for 10 s. Total protein contents from homogenates were analyzed using the acid bicinchoninic method (following manufacturer’s instructions; Pierce^TM^ BCA Protein Assay Kit) and total protein content was adjusted to 6 µg/µL in each sample. Then homogenates were mixed 1:1 with loading Laëmmli buffer. Protein samples (40 μg) were separated by 10% SDS-PAGE and transferred to nitrocellulose membranes (3 MM Whatman). Immunoblot analyses were performed by using the antibodies: anti-SIRT1 (rabbit polyclonal, 1:1000 dilution, Merck-Millipore; Cat. #07-131); anti-RBAP48/46 (rabbit polyclonal, 1:1000 dilution, Cell Signaling; Cat. 4633S); anti-NF-κB p65 (rabbit polyclonal, 1:1000 dilution, Santa Cruz; Cat. sc-372); anti-NF-κB p65 (acetyl K310) (rabbit polyclonal, 1:1000 dilution, Abcam; Cat. ab19870); anti-β-actin (mouse monoclonal, 1:10,000 dilution, Sigma-Aldrich; Cat. A1978). Secondary HRP-linked antibodies consisted of anti-rabbit IgG (goat polyclonal, 1:5000 dilution, Cell Signaling; Cat. #7074) and anti-mouse IgG (horse polyclonal, 1:5000 dilution, Cell Signaling; Cat. #7074). Proteins were detected using the ECL Western Blotting Detection Reagents (Amersham). The chemiluminescence bands were detected by exposure to photographic films (Hyperfilm Amersham) and digitalized with a GS-800 scanner, and the integrated optic density was analyzed with the QuantityOne software (Bio-Rad). Each sample was analyzed at least three times in different gels and membranes were reproved for β-actin, which was used for protein normalization. Mean values were expressed as percentage of immunoreactivity with respect to the young control group.

### 2.5. Statistics

Results are expressed as mean ± SEM. Two-way ANOVA was used for statistical evaluation of behavioral results in each experimental group throughout the entire period of treatments, using the Bonferroni post-hoc test for pairwise statistical comparisons. One-way ANOVA was used for other statistical evaluations, followed by the Tukey post-hoc test. The Pearson correlation coefficient was used to analyze associations between behavioral test results and neurochemical analysis in hippocampus and striatum as well as hippocampal protein levels. Level of significance was set at *p* ≤ 0.05. Data were analyzed with the program GraphPad Prism, version 6.0.

## 3. Results

### 3.1. Old Rats’ Cognitive Abilities Evolution during Chronic Treatment with Cathechin and Polyphenon-60

**Figure 1:**
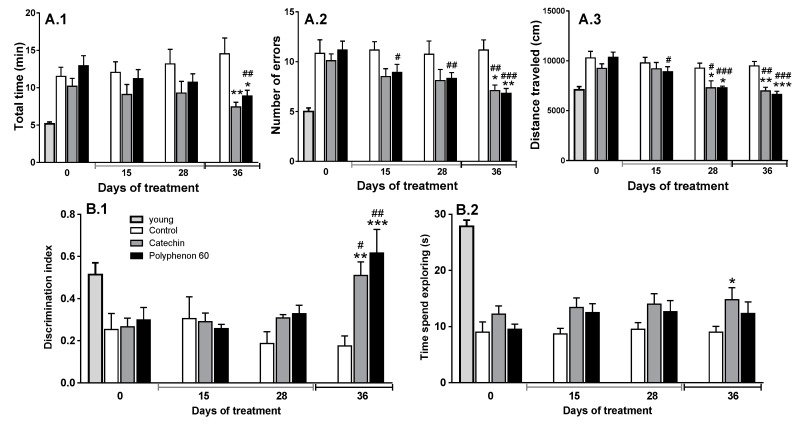
Evolution of spatial working memory and episodic memory of old rats (18 months) assessed during the chronic polyphenon-60 and catechin treatments. (**A**) Spatial working memory analysis through radial test. Bars represent the mean ± SEM of: time spent to complete the test in minutes (A.1), number of errors committed during the tests (A.2), and distance travelled during the tests in cm (A.3). (**B**) Episodic memory analysis through novel object recognition test. Bars represent the mean ± SEM of: discrimination index during tests (B.1), calculated by subtracting the time (in seconds) exploring the familiar object with respect to the time exploring the novel one and dividing by the total time exploring any object; and total time spend exploring both equal objects during familiarizing phase (in seconds, B.2). Animals were treated with catechin (*n* = 5; dark grey bars) or polyphenon-60 (*n* = 6; black bars) (20 mg/kg, i.p., for 28 days; followed by 40 mg/kg, i.p., during 7 additional days; both treatments). Control animals received 1 mL/kg of corn oil for 40 days (i.p.; *n* = 6; white bars). Young animal group (light grey bar) was used as reference to compare with old animals (*n* = 6). Two-way ANOVA followed by Bonferroni post-hoc test was used for statistical analysis. * *p* < 0.05, ** *p* < 0.01, *** *p* < 0.001 when comparing treated groups with control group at the same time point. ^#^ *p* < 0.05, ^##^ *p* < 0.01, ^###^ *p* < 0.001, when comparing data within each experimental group with respect to the initial data. Young animals were significantly different from all old rat groups when compared with the chronic treatments start for all parameters analyzed (one-way ANOVA followed by Bonferroni post-hoc test, *p* < 0.05 at least; comparison not indicated in the graph).

Weight of animals was not modified during chronic treatments and there were no significant differences in rat weight between groups (data not shown; the average weight of animals was 640 ± 5 g; *n* = 16). In contrast, some tendencies to improve cognitive abilities were observed after 28 days of treatment with catechin or polyphenon-60 (20 mg/kg, i.p. daily), but the most observed effect was not statistically significant (Figure 1). Thus, animals showed a tendency to reduce time needed to visit the eight arms in the radial arm test and errors committed; the error committed by the polyphenon-60 treated group was the only significant difference detected when compared with the old control group (Figure 1A).

Additionally, both treated groups showed a reduction in the distance walked along the radial arm test, suggesting an increase in efficiency in performing this test (Figure 1A). Furthermore, a similar effect was observed for the evolution of the novel object recognition test results. After 28 days of treatment, animals showed greater discrimination of the novel and familiar objects, showing as an increase in the discrimination index value, which was not statistically significant (Figure 1B). Since differences between groups were not statistically significant, we decided to extend the treatments, increasing the dose (up to 40 mg/kg, i.p. daily) for an additional week. After this period, animals showed a clear improvement in spatial working memory (showed by a reduction in the time needed to visit the eight arm and in the number of errors committed in the radial arm test; Figure 1A) and in episodic memory (showed by an increase in the discrimination index, Figure 1B) when compared with the old control group chronically treated with vehicle. These old animals chronically treated with catechin or polyphenon-60 showed similar results to those obtained by young animals in all radial maze parameters analyzed and for discrimination index in the novel object recognition test (Figure 1).

Motor coordination was analyzed using the rotarod device concomitantly with the already mentioned behavioral tests. No significant changes were observed in motor coordination in old animals. Animals remained on rotarod for a mean time of 19.46 ± 5.3 s, with no significant changes between the different experimental groups through any chronic treatment. Young animals remained on rotarod for 37.23 ± 2.9 s (data not shown).

### 3.2. Effect of Chronic Catechin and Polyphenol-60 Treatments in Old Rats on Monoamine Synthesis and Metabolism in Hippocampus and Striatum

**Figure 2:**
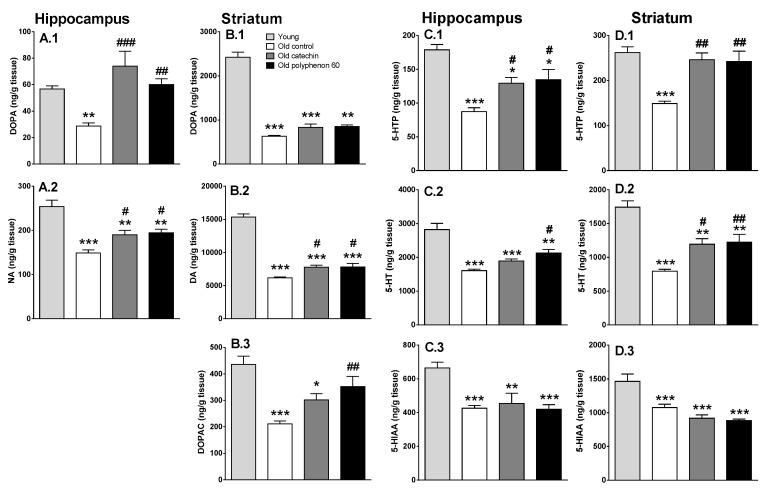
Effect of chronic polyphenon-60 and catechin treatments in catecholaminergic and serotonergic systems in hippocampus and striatum of old rats (18 months) and comparison with young rats (3 months). Catecholaminergic systems (**A** and **B** graphs): bars represent (mean ± SEM in ng/g of wet tissue) DOPA accumulation (during 30 min after decarboxylase inhibition) and NA or DA tissue content in hippocampus and striatum, respectively. DOPAC metabolite levels in striatum are also represented. Serotonergic systems (**C** and **D** graphs): bars represent (mean ± SEM in ng/g of wet tissue) 5-HTP accumulation (during the 30 min after decarboxylase inhibition), 5-HT tissue content, and 5-HIAA metabolite levels in hippocampus and striatum. Animals were treated with catechin (*n* = 5; grey bars) or polyphenon-60 (*n* = 6; black bars) (20 mg/kg, i.p., for 28 days; followed by 40 mg/kg, i.p., during an additional 7 days; both). Control animals received 1 mL/kg of corn oil for 40 days (i.p.; *n* = 6; white bars). Young animal group was used as reference to compare with old animals (*n* = 6). Two-way ANOVA followed by Tukey post-hoc test was used for statistical analysis. * *p* < 0.05, ** *p* < 0.01, *** *p* < 0.001 when compared with the young group. ^#^ *p* < 0.05, ^##^ *p* < 0.01, ^###^ *p* < 0.001 when comparing the treated old animals with the old control group.

Brain monoaminergic system decline occurs during aging, which has been related to the deterioration of cognitive abilities since brain regions related to cognitive functions show a major decline. For this reason, the monoaminergic system status in the hippocampus and striatum (two important brain regions in different cognitive functions) was analyzed. Old control rats showed a characteristic reduction in DOPA and 5-HTP accumulation in the hippocampus and striatum (Figure 2), indicating reduced activities of TH and THP2, respectively, due to aging. This effect was also accompanied by a reduction in total content of NA in hippocampus, DA in striatum, and 5-HT in hippocampus and striatum. Additionally, a marked reduction in metabolite levels (DOPAC level in striatum and 5-HIAA levels in hippocampus and striatum) was observed (Figure 2). HPLC method did not allow detection of any NA metabolite.

In turn, chronic treatments with polyphenon-60 and catechin (20 mg/kg daily for 28 days followed by 40 mg/kg for 7 days, i.p.) induced a monoaminergic systems improvement in old animals. Both chronic treatments increased TH activity in hippocampus (measured as DOPA accumulation in this brain region) reaching similar levels to those of young animals (Figure 2A.1). Consequently, total NA content in hippocampus also increased but at a lower level (Figure 2A.2). Since metabolites from NA could not be detected, it cannot be investigated whether this difference between the effect on synthesis and on the total content of NA in hippocampus can be due to a recent use of the neurotransmitter. Regarding dopaminergic system in striatum, and although a slight protective effect due to chronic treatments is intuited, it did not reach statistical significance in many cases. In this sense, both chronic treatments induced a small increase in DOPA accumulation in this brain region (around 30%–35%) but this did not reach statistical significance (Figure 2B.1). Additionally, a significant increase in total dopamine content was also induced (25%–26% increase) but at a low degree (Figure 2B.2). A small increase in the metabolite DOPAC was also observed (but only the polyphenon-60 effect reached statistical significance), suggesting an increase in dopaminergic neurotransmission (Figure 2B.3).

Both chronic treatments also increased TPH activity (measured as 5-HTP accumulation) in hippocampus and striatum (indicating TPH-2 activity). This time, the effect was more prominent in striatum where treated groups reached similar levels to young animals (Figure 2C.1,D.1). Consequently, total serotonin content also increased, again with higher effect in striatum (Figure 2C.2,D.2). In turn, no significant effect was observed in serotonergic neurotransmission in these brain regions analyzed, since no differences were observed in 5-HIAA metabolite levels in treated groups compared with old control group (Figure 2C.3,D.3).

Interestingly, statistically significant and negative correlations have been observed between radial maze results obtained at the end of the chronic treatments (36 days of treatment) and the monoaminergic neurochemical analysis in both brain regions from old animals (total neurotransmitter level, precursors accumulation, and metabolite content; except for 5-HIAA in striatum), indicating a positive correlation between monoaminergic systems recovery and the spatial working memory improvement (Table 1). On the other hand, a positive correlation between the discrimination index obtained during the novel object recognition test and neurochemical parameters is observed, although it is not as robust (Table 1). It is worthy to mention that better positive correlations are obtained when raw data are used instead of discrimination index; e.g., time expended exploring a novel object (data not shown) or time expended exploring the novel object minus the time expended exploring the familiar one (Table 1).

### 3.3. Effect of Chronic Catechin and Polyphenol 60 Treatments in Old Rats on SIRT1, RBAP46/48, and NF-κB Immunoreactivity in Hippocampus

SIRT1 and RBAP46/48 protein levels in hippocampus were measured since these proteins usually show a marked age-related reduction in hippocampus and have been related to the age-related decline in cognitive functions.

**Figure 3:**
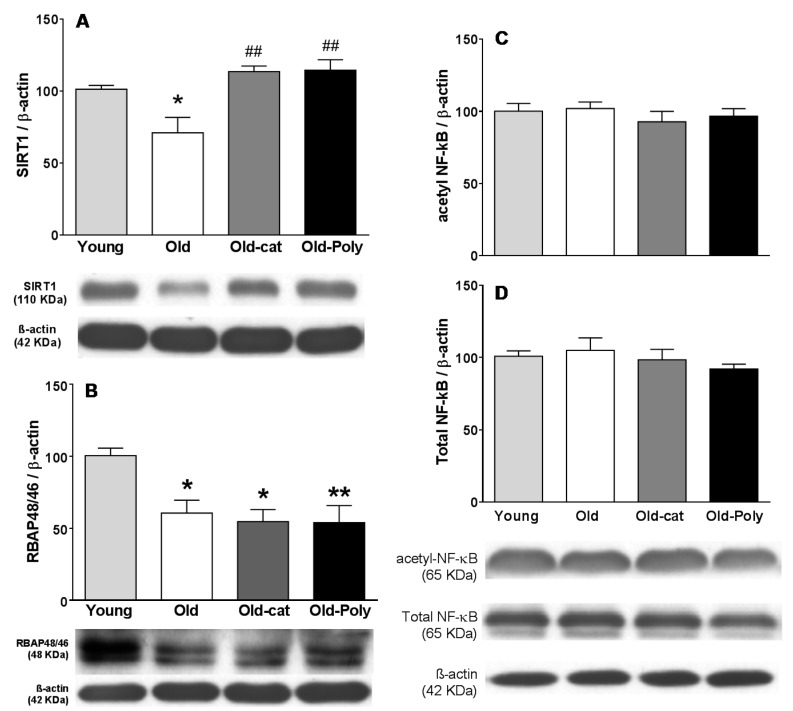
Effects of chronic polyphenon-60 and catechin treatments in hippocampal levels of SIRT1, RBAP46/48, and NF-κB in old rats (18 months) and comparison with young rats (3 months). Western blot analysis of hippocampal levels of total SIRT1 (**A**), RBAP46/48 (**B**), and acetylated (K310) and total NF-κB forms (**C** and **D**). Protein levels were normalized to β-actin content and each sample was analyzed in three different membranes. Bars represent mean ± SEM of protein levels expressed as percentage relative to the young group. Representative immunoblots are shown below graphs. Animals were treated with catechin (*n* = 5; grey bars) or polyphenon-60 (*n* = 6; black bars) (20 mg/kg, i.p., for 28 days; followed by 40 mg/kg, i.p., during an additional 7 days; both). Control animals received 1 mL/kg of corn oil for 40 days (i.p.; *n* = 6; white bars). Young animal group was used as reference to compare with old animals (*n* = 6). One-way ANOVA followed by the Tukey post-hoc test was used for statistical analysis. * *p* < 0.05, ** *p* < 0.01 when compared with young animals. ^##^ *p* < 0.01 when comparing treated groups with old control group.

Results from the present work confirmed that hippocampal levels of both proteins were reduced in old rats when compared with young ones (Figure 3A,B). In contrast, SIRT1 protein levels from old rats chronically treated with polyphenol-60 or catechin were significantly higher than in the old control rats, reaching close values to those of the young animals (Figure 3C). A significant correlation has been obtained between hippocampal SIRT1 protein level and cognitive performance at the end of chronic treatment in old animals (Table 1; a negative correlation with the time used to complete the radial maze test, and a positive correlation with the differences between time expended exploring the novel object and time expended exploring the familiar one during the novel object recognition test). However, in the opposite way, RBAP46/48 remained at a lower level in the chronically treated old animals (Figure 3D).

Surprisingly, old animals did not show changes in hippocampal NF-κB levels when compared with young animals, both for total NF-κB levels and for its acetylated form (Figure 3C,D). Consequently, no changes in hippocampal levels of NF-κB protein forms were detected in treated animals.

## 4. Discussion

Green tea extract is a widely consumed beverage related to several beneficial health effects due to its polyphenolic content. Most of its therapeutic potential has been associated to age-related diseases [16,19,41,42], in which oxidative stress and inflammaging have been identified as the leading underlying molecular mechanisms; mainly for brain alterations [16,25,27,43,44,45]. Indeed, like other polyphenolic compounds, the major polyphenolic compounds found in green tea extracts are catechins, which possess antioxidant and anti-inflammatory properties. Additionally, catechins can cross the blood–brain barrier, reaching the brain tissue, which allows their neuroprotective effects. In this way, several epidemiologic studies have shown that chronic green tea consumption had protective effect on age-related neurodegeneration and can improve brain function (for review see [10,27]). Although the main criticism for its therapeutic use, not only for the green extract polyphenols but also for all other dietary polyphenolic compounds, is their low bioavailability [46,47]. In this regard, different aspects must be taken into account that strengthen their potential therapeutic use (for example, some metabolites related to these compounds have therapeutic properties, among others) [48,49].

The results of the present work show a beneficial effect of these polyphenolic compounds on brain function, analyzed through cognitive tests. Animals showed a better performance in the eight-arm radial maze, indicating a better visuo-spatial working memory, and an increased episodic memory, analyzed through the novel object recognition test. Interestingly, chronic exposure to polyphenolic compounds is needed to produce a cognitive improvement (no acute or short-term effects were detected), suggesting a long-term adaptive mechanism for polyphenol neuroprotective effects, rather than an acute or fast effect.

Additionally, results from the behavioral tests did not evidence differences in anxiety or exploratory drive by effect of the chronic treatments (mainly assessed in the open field during the novel object recognition test; data not shown). This support the idea that the cognitive improvement after polyphenol treatments was due to the enhancement of memory and learning capacities but not as a consequence of other factors that could influence the performance of the behavioral test. On the contrary, polyphenol exposure did not recover the deterioration in motor coordination due to aging. Thus, rotarod performance did not change in old animals after chronic treatments, suggesting that the recovery in brain function is mainly related to memory and not to motor coordination, despite the fact that it did not affect the animal performance on the behavioral tests. Thus, reduction in distance walked along the radial maze test can be due to the better performance of animals during this test (less time expended to complete the test and less errors committed). These effects are in the same line with other reported studies linked to chronic treatments with other polyphenols or other antioxidant molecules [5,6,24,50]. These current results are related to the mild effects that chronic polyphenol treatments produce on dopaminergic systems in striatum, whose recovery is not as strong or evident as it is in other monoaminergic systems analyzed [5,6,24,39]. In turn, the observed cognitive function improvements are in good correlation with the recovery of noradrenergic system in hippocampus and serotonergic system in hippocampus and striatum after chronic polyphenol treatments. In fact, aging induces a marked decline of monoaminergic systems that has been linked to the typical age-associated cognitive deterioration [5,6,24,36,51,52]. Results from the present work showed a marked age-associated monoaminergic decline in hippocampus and striatum, since a marked decline in dopamine, noradrenaline, and serotonin syntheses rate (measured as DOPA and 5-HTP accumulation after aromatic amino acid decarboxylase inhibition) was observed. These synthesis rate reductions were probably a leading cause that produced a marked reduction in total neurotransmitters content (NA in hippocampus, DA in striatum, and 5-HT in both regions), thus contributing to the reduction in their metabolite levels in both regions. Loss of monoaminergic neuron axon terminals in these regions could also be a reason for the observed monoaminergic decline. Oxidative stress, as well the concomitant low grade neuroinflammation, seems to be one of the key effects producing neurodegeneration during normal aging [53,54,55]. Chronic exposure to a mixture of polyphenols from green tea (green tea extract polyphenon-60) or only to catechin protected the monoaminergic systems, but also seemed to recover them from the age-associated decline. The observed effects differed in both brain regions studied and in the monoaminergic system analyzed, but there were no differences between both tested treatments. Regarding catecholaminergic systems, a strong protective effect was observed in TH activity of hippocampus, even though only with little non-significant tendency occurring in the striatum. In hippocampus, NA synthesis (measured as hydroxylase enzyme TH activity) in old animals chronically treated with catechin or polyphenon-60 shows similar values to those observed in young rats. In this sense, it has been shown that antioxidant molecules protect TH from an oxidative stress status [54,55]. The increase in NA synthesis was correlated with a significant increase in total NA content in this region. By contrast, the TH activity in striatum was not affected by treatments; only slight (but not significant) tendencies to increase DOPA accumulation and DA content were found with respect to the old control animals. It is worth mentioning that these data are in line with previous results from our group regarding neuroprotective effects from other polyphenolic compounds [5,6]. At the same time, an improvement in serotonergic systems in the old animals after chronic treatments with polyphenols was observed in both brain regions. After chronic treatments with polyphenon-60 and with catechin, TPH activity (measured as 5-HTP accumulation during 30 min of aromatic amino acid decarboxylase inhibition) and total content of 5-HT were also increased in both brain regions. Thus, as described previously for other polyphenolic compounds [5,6], catechin and polyphenon-60 modulate monoamine neurotransmitters in key brain regions by contributing to the cognitive improvement in the old rats, probably by protection of the limiting enzymes of their synthesis, although additional molecular mechanisms should be considered.

Several molecular mechanisms have been postulated to underlie the beneficial effects of polyphenol on brain function, among them, epigenetic mechanisms seem to play a relevant role also for green tea polyphenols [26,56]. In this regard, proteins that modulate histone acetylation pattern could be responsible of many of these neuroprotective effects. SIRT1 has received a great deal of attention, believed to be a modulator of several neuroprotective effects in different therapies and having an important role in memory regulation as well as in other cognitive abilities [25,57]. A marked reduction in hippocampal SIRT1 levels has been described during aging and different neuroprotective strategies (including treatment with different polyphenolic compounds) have been reported to revert this reduction, pointing out this protein as a mediator of their neuroprotective effects [6,58,59,60]. Results from the current work showed that chronic exposure to green tea extract or to catechin recovered levels of SIRT1 proteins in old animals reaching values similar to those found in young animals. This result reinforces the relevant role of SIRT1 protein as one of the responsible agents in the neuroprotective effects of these therapies improving memory. In vivo, the protective effect from these therapies in brain SIRT1 levels seems to be mainly due to a protective effect against an oxidative status. Thus, Yamakuci et al. (2008) described that oxidative stress reduced SIRT1 mRNA levels inducing an inhibition of SIRT1 expression. In addition, cysteine residues from SIRT1 were vulnerable to oxidation affecting both the activity of SIRT1 and protein degradation by the proteasome (for review see [2]). Therefore, polyphenols may protect SIRT1 enzyme due to their antioxidant properties and, in turn, modulate proteins affected by SIRT1 activity. Apart from histones, SIRT1 also deacetylates other proteins, modulating their activity [61]. Among them, one key protein for inflammatory responses modulation is NF-κB. Thus, SIRT1 deacetylates the lysine at position 310 from p65 subunit, producing a prolonged activation when it is activated by external signaling; this has been related to an increase in inflammatory responses [61]. However, no changes were detected in the total hippocampal levels of p65 or in its acetylated form in any animals from the present study (neither in young nor in old ones). However, these results did not rule out a deregulation of NF-κB signaling during aging and after polyphenol exposure, due to its complex regulation and/or cell type specificity (for review see [62,63]). The possible role of RBAP46/48 in the protective effects of polyphenol treatments has been investigated; this is another protein that modulates histone acetylation patterns also associated with the age-related memory loss [34,35]. In this case, an age-associated reduction in hippocampal RBAP46/48 protein levels was corroborated by the current results, but polyphenol treatments were not able to reverse this reduction, as occurred for hippocampal SIRT1 protein. Thus, RBAP46/48 seemed not to have a relevant role in neuroprotective effects of polyphenon-60 or catechin.

A final consideration should be highlighted regarding neuroprotective differences between polyphenolic compounds. Although no great differences seem to exist between different polyphenolic compounds, huge research efforts have tried to attribute the main protective effects to particular polyphenolic compounds (e.g., for resveratrol) [5,6]. Regarding therapeutic potential of green tea extracts, most works have focused on its main component, epigallocatechin gallate, which constitutes around 50%–65% of the total content. Catechin represents only around 1%–4% of most green tea extract, whereas total polyphenolic content of polyphenon-60 is around 80%–90% [48,64,65,66]. Other works also show neuroprotective effects for catechin, in which oxidative stress counteraction has been postulated as the main underlying mechanism [30,67]. 

## 5. Conclusion

Results from the present work adds useful evidence indicating that the consumtion of green tea allows delaying cerebral senesce and, therefore, improving life quality in the elderly. In addition, these results, together with other evidences in the literature, seem to indicate that the neuroprotective effect of all these foods or beverages rich in polyphenols is not exclusive to one polyphenolic compound; and that the important aspect to consider in diets is the total polyphenolic content. In this sense, green tea extracts are an important source of polyphenols.

## Figures and Tables

**Table 1:** Correlation analysis between behavioral results at the end of the chronic treatments and neurochemical/protein levels detected in old animals.

	Radial Maze		Novel Object Recognition	
	Time	Errors	D.I.	Novel – Fam.
**Hippocampus**				
NA	−0.67 **	−0.8 ***	0.39	0.64 **
DOPA	−0.47*	−0.59 *	0.44	0.48 *
5-HT	−0.61**	−0.76 ***	0.50 *	0.6 *
5-HTP	−0.48*	−0.48 *	0.11	0.72 **
5-HIAA	−0.11	−0.09	0.02	−0.07
**Striatum**				
DA	−0.55 *	−0.69 **	0.6 *	0.7 **
DOPA	−0.38	−0.68 **	0.34	0.42
DOPAC	−0.25	−0.51 *	0.53 *	0.43
5-HT	−0.6 *	−0.72 **	0.22	0.75 **
5-HTP	−0.62 **	−0.66 **	0.43	0.75 **
5-HIAA	0.44	0.37	−0.35	−0.46
**Hippocampal Protein Level**				
SIRT1	−0.82 ***	−0.44	0.44	0.55 *
RBAP49/46	0.19	0.05	−0.33	−0.46
Total NF-κB	0.24	0.21	−0.38	−0.28
Acetyl-NF-κB	−0.16	−0.22	−0.26	0.04

Pearson correlation coefficients between indicated parameters are represented; *p*-values are indicated as: * *p* < 0.05, ** *p* < 0.01, *** *p* < 0.001.
